# Combined ablation of atrial fibrillation and minimally invasive mitral valve surgery: a case report

**DOI:** 10.1186/1749-8090-5-79

**Published:** 2010-10-11

**Authors:** Hironori Izutani, Masahiro Ryugo, Fumiaki Shikata, Masashi Kawamura, Tatsuhiro Nakata, Toru Okamura, Takumi Yasugi, Mitsugi Nagashima, Kanji Kawachi

**Affiliations:** 1 Department of Cardiovascular & Thoracic Surgery, Ehime University Graduate School of Medicine, Ehime, Japan

## Abstract

A partial lower inverted J sternotomy and an extended transseptal incision provide excellent exposure for minimally invasive mitral valve surgery. However, the extended trasnsseptal incision causes dividing the sinus node artery, which may result in conduction system disturbance and need for permanent pacemaker implantation. Therefore, there is a challenge in the patient who requires concomitant ablation for atrial fibrillation because of possible conduction system disturbance caused by extended transseptal incision. We describe a new strategy for combined ablation of atrial fibrillation with minimally invasive cardiac surgery by a transseptal approach to the mitral valve through a partial lower sternotomy incision. Cryoablation was performed using a T-shaped cryoprobe with a lesion set of pulmonary vein isolation and ablation of the left and right isthmus in performing mitral annuloplasty, tricuspid annuloplasty, and atrial septal defect closure through a limited sternotomy incision. This technique might minimize possible conduction system disturbance and provide good surgical result for the patients who undergo mitral valve surgery and ablation of atrial fibrillation.

## Introduction

Minimally invasive cardiac surgery with partial sternotomy for valvular heart disease has been performed for more than a decade. A partial lower sternotomy and an extended transseptal incision provide excellent exposure for minimally invasive mitral valve surgery [1,2]. We have experienced sixty minimally invasive surgeries with partial sternotomy since 2004. This approach provides excellent results in less pain, less blood loss, lower rate of wound complications, shorter length of hospital stay, and excellent cosmetics. However, there is a challenge in the patient who requires combined ablation of atrial fibrillation because of possible conduction system disturbance caused by the extended transseptal approach. We carried out cryoablation in three patients for chronic atrial fibrillation with good clinical results using a T-shaped cryoprobe with a lesion set of pulmonary vein isolation and ablation of the left and right isthmus in performing minimally invasive mitral valve surgery. We describe our technique for a creation of a lesion set for ablation of atrial fibrillation using the transseptal approach to the mitral valve through a partial lower sternotomy incision.

## Case report

**Figure 1:**
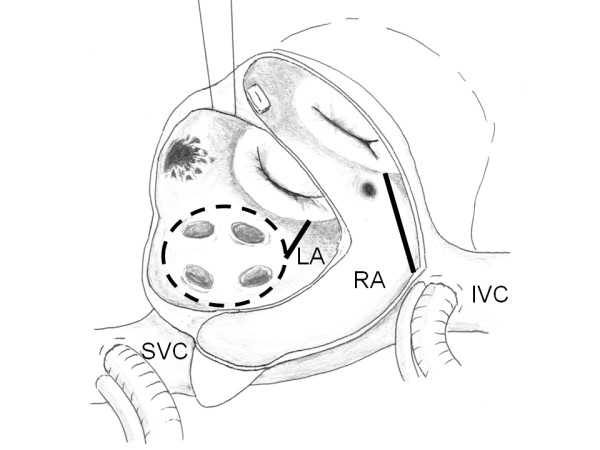
**Schematic view of right atrium (RA) and left atrium (LA) through a transseptal approach to the mitral valve**. Creation of a cryoablation lesion set for atrial fibrillation ablation: combination of pulmonary vein isolation (dashed lines) and ablation of the left and right isthmus (solid lines). (SVC = superior vena cava; IVC = inferior vena cava.)

**Figure 2:**
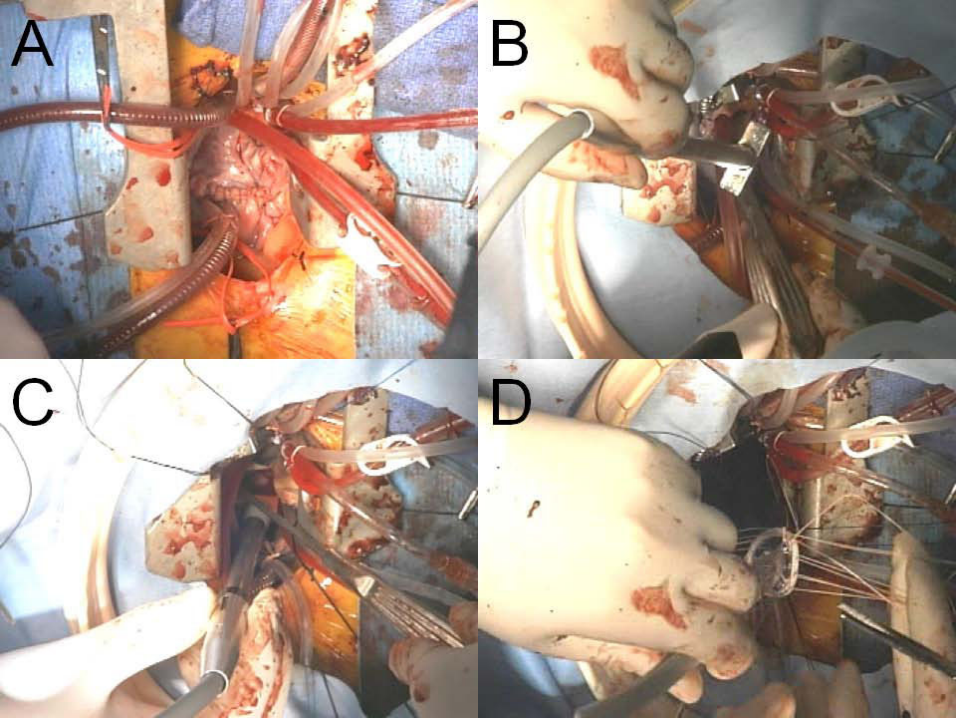
**Intraoperative photographs showing a lower inverted J partial sternotomy incision with cardiopulmonary bypass (A), a T-shaped cryoprobe (B) used for the lesion set through the small surgical field (C), and insertion of a mitral annuloplasty ring (D)**.

A 72-year-old man with a history of chronic atrial fibrillation recently experienced palpitation and dyspnea on effort. His echocardiography showed an atrial septal defect, moderate mitral regurgitation, moderate tricuspid regurgitation, and slightly reduced left ventricular function with an ejection fraction of 49%. His cardiac catheterization studies showed the Qp/Qs of 3.46 and mean pulmonary pressure of 23 mmHg. The patient was recommended to undergo mitral valve repair, tricuspid valve repair, atrial septal defect closure, and ablation of atrial fibrillation. A seven centimeter midline chest skin incision was made. The sternal saw was used to perform partial sternotomy from the right second intercostal space down to the xyphoid. A 7 mm soft-flow aortic cannula was placed on the ascending aorta. Bicaval venous cannulation was performed with 22 Fr cannulas. The patient was placed on cardiopulmonary bypass with vacuum assisted venous return. An aortic cross-clamp was placed and cardiac arrest was achieved by cold blood antegrade cardioplegia. Snaring down the vena cavas, the right atrium was opened longitudinally. A retrograde cardioplegic catheter was placed into the coronary sinus for intermittent cardioplegia administration. The incision was extended to the left of the right auricle toward the left atrium posteriorly. There was a 2 cm-length foramen ovale type atrial septal defect. The residual foramen ovale was cut at the middle then the incision was extended toward the right atriotomy incision and the dome of the left atrium. The mitral valve was exposed by a transseptal approach (Figure 1). Left side ablation was performed by cryoablation at -60°C for 2 minutes on each point in order to isolate the pulmonary veins. Cryoablation was also applied on the left and right atrial isthmus. The lesion set was created in 20 minutes. The left atrial appendage was closed by sewing over its orifice with a 4-0 polypropylene running suture. Mitral annuloplasty was carried out to plicate the posterior annular dilatation with a 24 mm Edwards Physio-ring (Edwards Lifesciences, Irvine, CA). The left atrium and the atrial septum including the atrial septal defect were closed directly with sutures. Then tricuspid annuloplasty was performed with a 26 mm Edwards MC^3 ^(Edwards Lifesciences, Irvine, CA). The right atrium was closed and the aortic cross clamp was released. Intraoperative photographs were shown in Figure 2. Cardiac arrest time was 165 minutes. The heart beat started spontaneously with nodal rhythm. The surgery time was 316 minutes. The heart rhythm returned to normal sinus rhythm a day after the surgery. The patient recovered uneventfully and he was discharged home at the 10th postoperative day. He has maintained normal sinus rhythm for one year postoperatively without antiarrhythmic medication.

## Discussion

Several studies suggest that the extended transseptal approach carries an increased risk of early postoperative arrhythmias compared with the standard left atrial incision. The extended trasnseptal incision causes dividing the sinus node artery, which may result in conduction system disturbance and need for permanent pacemaker implantation [3]. Kumar and colleagues reported early postoperative prevalence of junctional rhythm in 38% of the patients who underwent the transseptal approach, with resolution of sinus rhythm in a certain proportion of patients [4]. Lukac and colleagues demonstrated a statistically significant difference in the occurrence of permanent pacemaker implantation for sick sinus syndrome between patients undergoing the transseptal approach and left atriotomy through the interatrial groove (6% versus 2.3%, respectively) [5]. On the other hand, Légaré and colleagues showed that there was no difference in the prevalence of postoperative arrhythmias and permanent pacemaker insertion among the approaches through left atrial dome, interatrial groove, and atrial septum in 131 patients [6]. We performed minimally invasive mitral valve surgery using the transseptal approach in 35 patients with preoperative sinus rhythm. Six patients developed junctional rhythm with or without bradycardia postoperatively, but there was no patients requiring permanent pacemaker implantation. The distribution of the sinus node artery was checked preoperatively by coronary angiography. We carefully extend the incision toward the dome of the left atrium to avoid injury of the sinus node artery in performing transseptal approach.

Gillinov and colleagues described a new technique for creation of a lesion set for atrial fibrillation ablation using the transseptal approach to the mitral valve through the minimally invasive partial sternotomy [7]. They successfully did ablation using a combination of bipolar radiofrequency and cryothermy. We made a lesion set of ablation of atrial fibrillation which was different from that of Gillinov's technique. Our technique consists of a combination of pulmonary vein isolation and ablation of the left and right atrial isthmus using cryothermy. It is based on a technique described by Sueda and colleagues [8]. They reported mid-term results of pulmonary vein isolation for the elimination of chronic atrial fibrillation. They showed excellent early results with the cumulative elimination rate of 70.2%. They commented that a requirement for a permanent pacemaker implantation was less frequent than that of standard MAZE procedure. They concluded that pulmonary vein isolation was effective and safe for surgical treatment of chronic atrial fibrillation.

## Conclusions

Our technique of a minimally invasive approach with a 7-cm skin incision and partial lower sternotomy can be used to perform mitral valve, tricuspid valve procedure, atrial septal defect closure, and atrial fibrillation ablation. Three patients underwent ablation of atrial fibrillation in minimally invasive mitral valve surgery with favorable results. Preoperatively, the present patient had chronic atrial fibrillation, and the other two had paroxysmal atrial fibrillation and mitral regurgitation without atrial septal defect. They maintained sinus rhythm at least six months postoperatively. However continued careful follow-up should be mandatory for confirming the usefulness of this technique.

## Consent

Written informed consent was obtained from the patient for publication of this case report and accompanying images. A copy of the written consent is available for review by the Editor-in-Chief of this journal.

## Competing interests

The authors declare that they have no competing interests.

## Authors' contributions

All authors read and approved the final manuscript.
